# Impact of Non‐Pharmaceutical Interventions Targeted at COVID‐19 Pandemic on Influenza Burden—A Systematic Review and Meta‐Analysis

**DOI:** 10.1111/irv.70301

**Published:** 2026-07-29

**Authors:** Laura‐Inés Böhler, Lisa Koeppel, Stefan Fabian Weber, Mary Gaeddert, Katharina Thielemann, Kerstin Glaser, Horeya M. Ismail, Ulrich Reinacher, Veronika K. Jaeger, Julia Böhnke, Antonia Bartz, Maja Pavic, Manuela Harries, Christina Kuczewski, Torben Heinsohn, Olga Hovardovska, Sin‐Yin Huei, Chao Xu, Cornelia Gottschick, Seba Contreras, Maciej Filinski, Maurizio Grilli, Berit Lange, RESPINOW Study Group, Claudia M. Denkinger

**Affiliations:** ^1^ Department of Infectious Disease and Tropical Medicine Heidelberg University Medical Faculty, Heidelberg University Hospital Heidelberg Germany; ^2^ Department of Parasitology Heidelberg University Medical Faculty, Heidelberg University Hospital Heidelberg Germany; ^3^ German Center for Infection Research (DZIF), Partner Site Heidelberg Heidelberg Germany; ^4^ Robert Koch‐Institut Berlin Germany; ^5^ Institute of Epidemiology and Social Medicine University of Münster Münster Germany; ^6^ Helmholtz Centre for Infection Research Department of Epidemiology Braunschweig Germany; ^7^ TI BBD German Centre for Infection Research (DZIF) Braunschweig Germany; ^8^ Institute for Medical Epidemiology, Biometrics and Informatics, Interdisciplinary Centre for Health Sciences Medical Faculty of the Martin Luther University Halle‐Wittenberg Halle (Saale) Germany; ^9^ Institute for the Dynamics of Complex Systems University of Göttingen Göttingen Germany; ^10^ Max Planck Institute for Dynamics and Self‐Organization Göttingen Germany; ^11^ Faculty of Information and Communication Technology Wrocław University of Science and Technology Wrocław Poland; ^12^ Medical Faculty Mannheim Heidelberg University Heidelberg Germany; ^13^ Translational Lung Research Center Heidelberg (TLRC), German Center for Lung Research (DZL) University of Heidelberg Heidelberg Germany

## Abstract

Seasonal influenza imposes a substantial global health burden. The COVID‐19 pandemic, accompanied by widespread non‐pharmaceutical interventions (NPIs), profoundly disrupted respiratory virus circulation, offering a unique opportunity to assess their broader epidemiological impact. This study aimed to quantify changes in influenza burden between the pre‐pandemic and intra‐pandemic periods. Within the RESPINOW project, we conducted a systematic review following PRISMA guidelines. Studies reporting absolute influenza case counts before and during the pandemic were included. Data extraction and quality assessment were performed using a modified NHLBI tool for before–after studies. Influenza cases were normalized by reporting period length, and relative changes were estimated using incidence rate ratios. Subgroup analyses explored age, setting, hemisphere, human development index, influenza transmission zones, WHO regions, and viral strains. Of 20,676 screened records, 115 studies from 98 countries were included. Globally, influenza incidence declined by −92% (95% CI: −94 to −90) following the onset of the pandemic. Reductions varied geographically, ranging from near‐elimination in several countries to more modest declines (e.g., South Korea: −61%, 95% CI: −79 to −26). Decreases were observed across all transmission zones and WHO regions, with descriptively larger reductions observed in high‐income countries (−96%, 95% CI: −98 to −94) than in low‐income settings (−82%, 95% CI: −88 to −73). Age‐specific declines appeared smaller among young children (< 6 years: −66%, 95% CI: −79 to −45) compared with adults aged 18–64 years (−80%, 95% CI: −90 to −63). Influenza A appeared to decline more strongly than influenza B. The COVID‐19 pandemic was associated with an unprecedented global reduction in influenza incidence. Data limitations and the need for robust epidemiological indicators highlight the importance of strengthened, integrated global surveillance to inform future pandemic responses.

## 1 Introduction

Influenza is an acute viral respiratory infection caused mainly by influenza A and B viruses, and it has long been recognized as a major global public health concern. Seasonal influenza epidemics are responsible for substantial morbidity and mortality worldwide. According to the World Health Organization (WHO), annual influenza epidemics result in an estimated 3–5 million cases of severe illness and between 290,000 and 650,000 respiratory deaths annually worldwide [1].

With the emergence of the COVID‐19 pandemic in early 2020, widespread implementation of non‐pharmaceutical interventions (NPIs) such as masking, physical distancing, school closures, international travel restrictions, and improved hand hygiene took place [2, 3]. The implementation and stringency of NPIs differed by country but generally included measures such as the prohibition of larger gatherings and social distancing, border closures and travel restrictions, school closures, isolation of symptomatic individuals and their contacts, and widespread lockdowns restricting all but essential internal movement [4].

After the implementation of COVID‐19‐related NPIs, global influenza activity fell to historically low levels [5, 6, 7]. The Global Burden of Disease (GBD) 2021 analysis showed that influenza‐associated lower respiratory tract infection deaths declined globally by approximately 71.8% compared with pre‐pandemic years since 1990, underscoring the indirect protective effects of COVID‐19 mitigation measures [8].

In summary, before COVID‐19, influenza represented a predictable but severe seasonal epidemic with a substantial global health burden. During the pandemic, influenza circulation and associated mortality declined dramatically likely due to NPIs, while the subsequent easing of restrictions has been accompanied by a resurgence with altered epidemiological patterns [9]. These shifts highlight the dynamic interaction between public health measures, viral transmission, and population immunity, and they underscore the importance of sustained global surveillance and vaccination efforts.

Drawing evidence from the SARS‐CoV‐2 pandemic years, this systematic review and analysis contributes to a broader body of work aimed at developing integrated models to simulate the transmission of respiratory infections and to assess the collateral effects of non‐pharmaceutical interventions, such as those pursued within initiatives like the RESPINOW modelling consortium [10]. Therefore, the objective of the work in this manuscript was to thoroughly explore the global effect of the COVID‐19 pandemic on influenza burden by quantifying the change in Influenza incidence between the pre‐pandemic and intra‐pandemic periods. Recognizing how NPIs targeted at COVID‐19 may impact transmission of other related respiratory pathogens, such as influenza, can provide insights for future pandemic response.

## 2 Methods

### 2.1 Search Strategy

**FIGURE 1:**
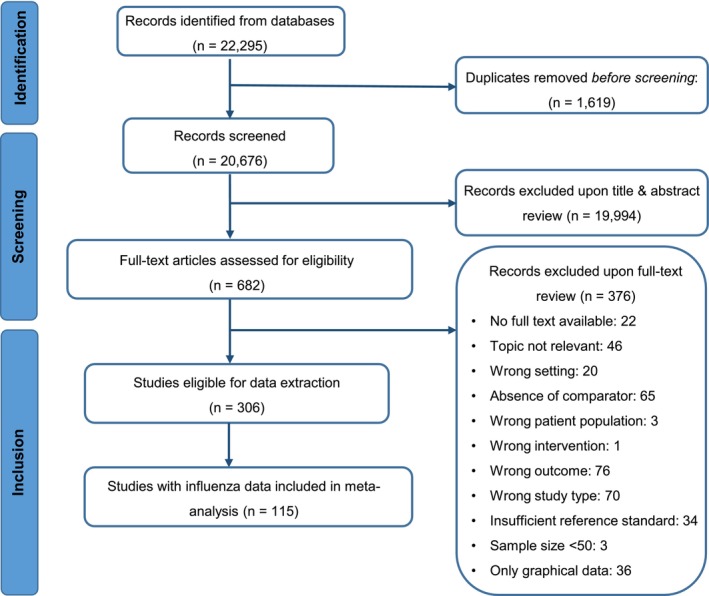
PRISMA flow diagram of study selection.

This meta‐analysis is part of a larger review including influenza, respiratory syncytial virus (RSV), and pneumococcal disease (see Figure 1). The results presented in this manuscript refer only to the influenza‐related portion. A literature search was conducted across PubMed, Cochrane Library, Google Scholar, Web of Science, MedRxiv/BioRxiv COVID‐19 preVIEW, and ScienceDirect using English synonyms for influenza (and also RSV, or pneumococcal disease—not addressed in this paper) and were combined using the Boolean OR operator and subsequently linked to English synonyms for SARS‐CoV‐2 with the Boolean AND operator; the full list of search terms is provided in Material S2. The literature search was performed on August 28, 2023. This review follows the PRISMA (Preferred Reporting Items for Systematic reviews and Meta‐Analyses) guidelines for reporting systematic reviews, see Checklist in Material S3 [11], and is registered on PROSPERO (Record ID CRD42022360015) [11].

#### 2.1.1 Inclusion and Exclusion

Studies reporting absolute and relative influenza case numbers, incidence, positivity rates, hospitalizations, mortality, and seasonal peak information comparing pre‐COVID‐19 pandemic and intra‐pandemic timepoints or seasons published after 2019 were included. No restrictions were applied to the pre‐pandemic study period, and the onset of the pandemic period was defined by the implementation of NPIs in the respective countries. The duration of the pandemic period was not predefined and was limited by the date of the systematic literature search. Only studies reporting data from single countries were included, and studies presenting multi‐country aggregated data were excluded. Cohort studies, cross‐sectional studies, surveillance studies, randomized controlled trials, and case–control studies were included. The following study types were excluded: (1) case reports, case series (< 50 cases), modeling studies, commentaries or editorials, and studies, which did not report primary data; (2) studies that analyzed special infection dynamics during pilgrimages, for example, the Haj; (3) studies that did not refer to the COVID‐19 pandemic as a comparator to pre‐pandemic periods; (4) studies focusing on in vitro analyses or animal studies. Furthermore, studies focusing on or including co‐infections/co‐morbidities or vaccination outcomes were also excluded, as this may have limited the possible conclusions on NPI impact on incidence.

#### 2.1.2 Screening and Data Extraction

Title and abstract screening and full text review were performed by two independent reviewers using the Covidence software [12]. Conflicts were resolved by discussion between the reviewers and a senior reviewer. Reasons for exclusion were recorded. Data extraction was performed for the included studies by one reviewer and checked by a second reviewer.

Data from England were merged under the United Kingdom. Taiwan was grouped under Eastern Asia for influenza transmission zones, as it was not listed separately. If age was not reported, a range of 0–120 years was assumed and for pediatric‐only data, an age range of 0–18 years was used. If no disease subtype was specified, cases were classified as influenza of unspecified subtype (A or B).

### 2.2 Risk of Bias Assessment

The quality assessment was conducted using a modified version of the NHLBI Quality Assessment Tool for Before–After Studies [13]. The questions were adapted to fit the review design, using Questions 1–3 and 5–7 from the original tool, and including two additional questions assessing data collection time range and stratification. The quality assessment tool is provided in Material S4. Two reviewers performed the assessment independently and any discrepancy was resolved by discussion.

### 2.3 Statistical Analysis

The objective of this statistical analysis was to quantify the change in Influenza incidence between the pre‐pandemic and intra‐pandemic periods.

To classify the extracted time periods as either *pre‐pandemic* or *during‐pandemic*, we used the onset of NPIs in each respective country as a reference point. Data for NPI onset was taken from the Oxford government response tracker [14]. Time intervals that ended before the NPI onset were classified as *before pandemic*, while those that began after the NPI onset were classified as *during pandemic*. In cases where a time interval overlapped the NPI onset, starting before but ending after, entries were excluded from the analysis. No post‐pandemic category was defined, as the COVID‐19 pandemic was ongoing at the time of the systematic literature search.

Influenza case data were available only in aggregated form over defined time intervals (e.g., monthly totals) and were extracted accordingly. To enable meaningful comparisons across studies, case numbers were normalized by the length of their respective reporting periods.

Under the necessary assumption of nearly equal population at risk, incidence rate ratios (IRRs) were calculated by dividing during‐pandemic time‐normalized case counts by the corresponding pre‐pandemic values. Data entries with zero cases in the pre‐pandemic period, which occurred in only a single study, were excluded, as relative change could not be computed. To account for the remaining cases of zero counts of events during the observation period, we added 0.5 to cell counts prior to time normalization (continuity correction). To avoid double counting, overlapping groups—such as data reported for both the total population and specific age groups—were deduplicated and the lowest appropriate level was chosen for each analysis.

For summary estimation, incidence rate ratios (IRRs) were log‐transformed. The mean and standard deviation were calculated on the log scale; 95% confidence intervals (CI) were derived assuming approximate normality, and results were exponentiated to obtain the time‐normalized mean IRR and corresponding CIs. To further investigate temporal trends across the pandemic period, we compared the pre‐pandemic time span separately with the years 2020, 2021, and 2022 to assess potential year‐specific differences in influenza incidence. Where sample size allowed, we also performed subgroup analysis with respect to the disease strain, the setting (hospital inpatients, outpatients), hemisphere, World Trade Organisation (WTO) classification (developed country, developing country, least developed country) [15], influenza transmission zone [16], WHO region [17], human development index (HDI) [18], country, and age groups (< 2, < 6, < 18, 18–64, ≥ 65, and ≥ 80 years).

## 3 Results

### 3.1 Search and Data Quality

The systematic search conducted as part of the RESPINOW project identified 20,676 records after deduplication for title and abstract screening. Of these, 682 articles underwent full‐text review, and 115 met the inclusion criteria for the influenza portion of the review (Figure 1).

**FIGURE 2:**
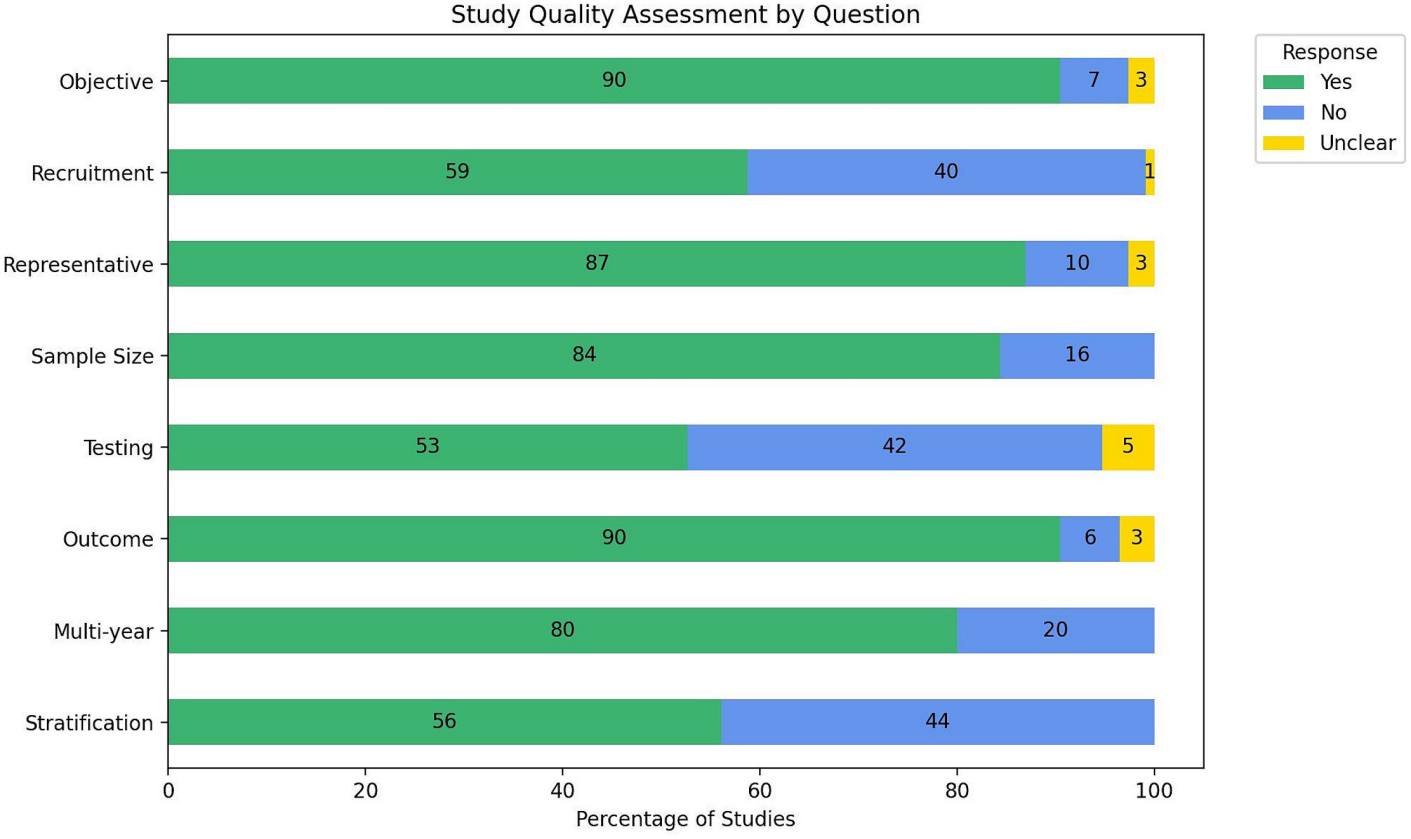
Summary of Quality assessment based on NHLBI criteria. Detailed questions are provided in Material S4.

The results of the quality assessment showed that most studies clearly described their study objectives, reported an adequate sample size, and indicated that the sample was representative of the target population (Figure 2). Moreover, outcome measures were prespecified in the majority of the included studies. In contrast, information on participant recruitment procedures and testing conditions was reported in only slightly more than half of the papers. Approximately 80% of the studies presented data spanning more than 1 year, and around half provided stratified analyses. Detailed results are presented in Material S5.

### 3.2 Overall Dataset and Geographic Strata

**FIGURE 3:**
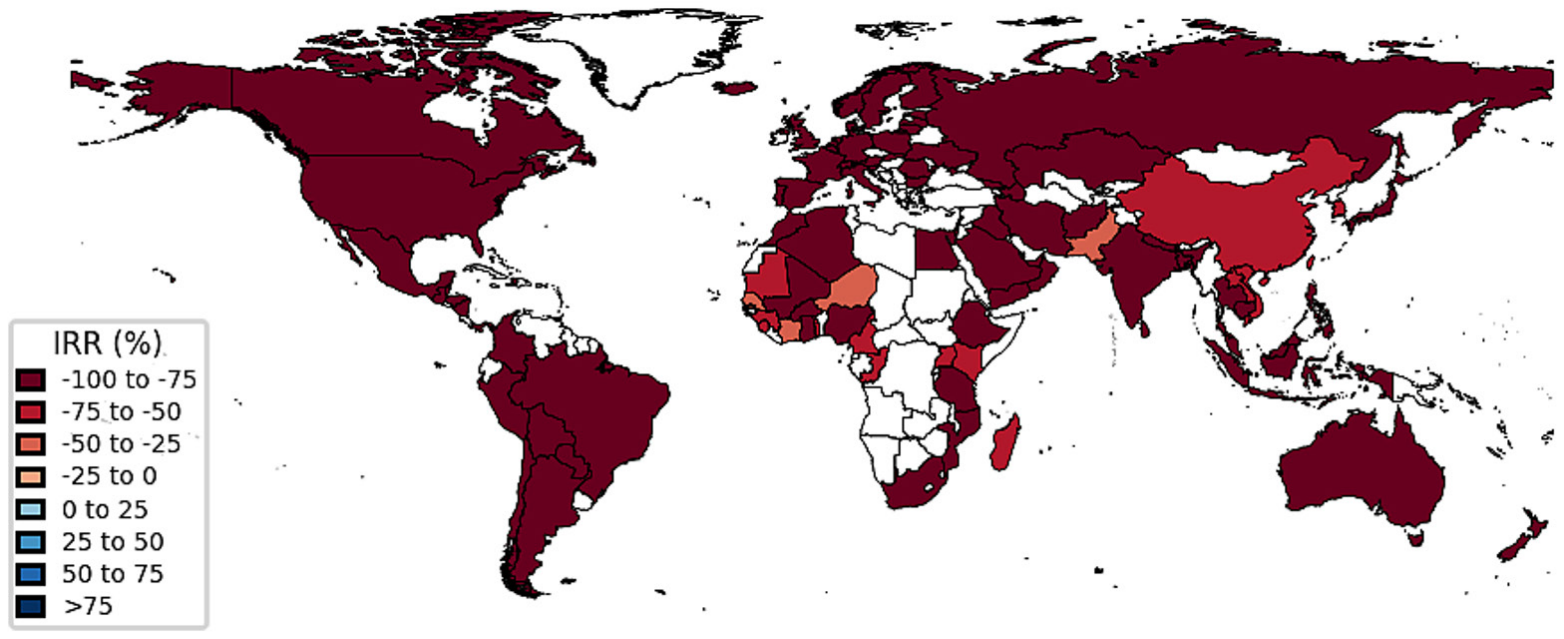
Spatial distribution of relative change in influenza incidence from the pre‐pandemic period to the full pandemic period. The white color denotes missing data.

Of the included studies, 44% were cohort studies and 52% were surveillance studies. Data were included from 98 countries (Figure 3). The countries contributing the most studies were China (*n* = 34), Japan and the United States (*n* = 11 each), and Australia (*n* = 10). Almost half of the papers (*n* = 65; 57%) reported age strata for individuals under 18 years, whereas only 24 (21%) papers provided data for children under 6 years. Sub‐strain information for influenza A and B was reported in 35 (30%) and 37 (32%) papers, respectively. The extracted raw data and detailed outcome summary results including stratifications can be found in Table S1.

The overall dataset showed a marked reduction (−92%; 95% CI: −94 to −90; *n* = 115) in influenza incidence between pre‐pandemic and intra‐pandemic time‐periods.

The incidence reduction ranged from countries virtually eliminating influenza incidence (e.g., Norway [*n* = 3], Singapore [*n* = 3], Switzerland [*n* = 2]: −99%) to others that showed a smaller decrease (e.g., South Korea: −61%; 95% CI: −79 to −26; *n* = 5), see Figure S10A, and country‐level data in Table S1.

Using the WHO influenza transmission zones, the same marked reductions can be observed (e.g., Northern Europe: −99%; 95% CI: −100 to −98; *n* = 7), while African zones showed moderate reductions (e.g., Western Africa: −66%; 95% CI: −78 to −48; *n* = 2 or Middle Africa: −67%; 95% CI: −73 to −60; *n* = 1) (Material S6). Clustering by WHO regions reveals a similar trend (Table S1).

Descriptive differences were observed between hemispheres, with reductions appearing larger in the Southern hemisphere (−97%; 95% CI: −98 to −94; *n* = 15) compared with the Northern hemisphere (−91%; 95% CI: −93 to −88; *n* = 102). Categorizing by WTO development status, “developed” status countries appeared to show larger reductions than those with “developing” or “least developed” status (developed: −96%; 95% CI: −97 to −94; *n* = 72, developing: −88%; 95% CI: −91 to −83; *n* = 44 or least developed: −82%; 95% CI: −88 to −73; *n* = 4). This is in line with the results considering the HDI (Materials S7 and S8 and Table S1).

### 3.3 Population‐Level

**FIGURE 4:**
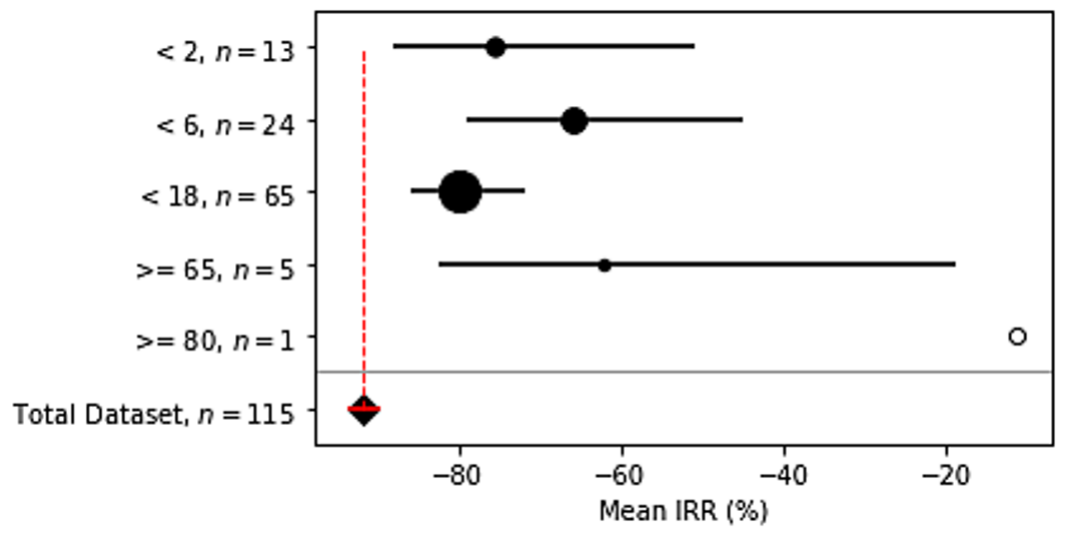
Forest plot of the relative change in influenza incidence for the full pandemic period compared to the pre‐pandemic period, stratified by age groups in years (y axis). The dot represents the mean IRR, the size of the dot the number of papers and the horizontal line the respective 95% confidence interval. An open circle indicates that data were reported by a single paper. Age categories are not mutually exclusive, and some data may contribute to multiple strata. The “Total Dataset” additionally includes studies that did not report age information or provided data not suitable for any of these strata, for example, for the full age range.

Considering the affected population by age groups, children (any age < 18 years) showed a similar (−80%; 95% CI: −86 to −72; *n* = 65) overall reduction to the “working age” adult group (18–64 years) (−80%; 95% CI: −90 to −63; *n* = 8). The retirement age group (≥ 65 years) displayed a more moderate reduction (−62%; 95% CI: −82 to −19; *n* = 5); see Table S1. Further examination of pediatric data suggested smaller reductions among children aged < 6 years (−66%; 95% CI: −79 to −45; *n* = 24) compared with those aged < 2 years (−76%; 95% CI: −88 to −51; *n* = 13). These sub‐strata of age groups do not include all papers due to the level of data aggregation and are provided in Figure 4. Influenza incidence stratified by hospital inpatient or outpatient setting showed a similar reduction (−82%; 95% CI: −89 to −71; *n* = 52) in inpatients compared to outpatients (−80%; 95% CI: −88 to −66; *n* = 10).

### 3.4 Strain‐Level

Some studies provided data on individual influenza strains. In general, reductions in incidence appeared greater for influenza A strains than for influenza B strains (A: −99%; 95% CI: −99 to −98; *n* = 35, B: −93%; 95% CI: −95 to −90; *n* = 37). Within the B strain influenza, Victoria strain appeared to show a similar reduction (−97%; 95% CI: −98 to **−**94; *n* = 5) to the Yamagata strain (−96%; 95% CI: −98 to −94; *n* = 5); see Figure S9 and Table S1.

### 3.5 Year‐By‐Year Comparisons

For individual year comparisons (pre‐pandemic vs. 2020, 2021, 2022), 66 (57%) articles provided data for 2020, 29 (25%) for 2021, and 6 (5%) for 2022. A summary for annual data is provided in Table S1; all statistics for individual years including all substrata are provided in individual tabs.

Overall, the greatest reduction in influenza incidence was observed in 2021 (−94%; 95% CI: −97 to −88; *n* = 29), whereas in 2022, an increase was observed (413%; 95% CI: −4 to 2648; *n* = 6). Due to the aggregation level and availability of articles, annual transmission zone dynamics could only be assessed for all years for Eastern Asia (−78%; 95% CI: −85 to −70; *n* = 33 in 2020; −83%; 95% CI: −95 to −45; *n* = 11 in 2021; 1277%; 95% CI: 132 to 8087; *n* = 33 in 2022) and South West Europe (−56%; 95% CI: −88 to 56; *n* = 10 in 2020; −98%; 95% CI: −99 to −93; *n* = 9 in 2021; −63%; 95% CI: −95 to 150; *n* = 3 in 2022).

Longitudinal analyses for substrata (e.g., inpatient/outpatient, age groups and other country‐level data) were restricted by the small number of available articles with data for all individual years and are provided in Table S1.

## 4 Discussion

Overall, these results showed a marked reduction in the incidence of influenza cases between the pre‐pandemic period and the period following the implementation of NPIs, with a median reduction of 92%. This marked decline can be attributed to the effectiveness of NPIs (e.g., social distancing, mask mandates, and school closures) in limiting the transmission of respiratory viruses beyond SARS‐CoV‐2.

Notably, influenza circulation did not cease entirely and residual transmission may reflect incomplete adherence to NPIs, local variations in NPI implementation, or ongoing low‐level circulation in a‐ or oligosymptomatic individuals. This finding supports the conclusion that NPIs have a broad suppressive effect on viruses with similar modes of transmission.

While most countries and regions experienced near‐complete suppression of influenza following the implementation of NPIs, the extent of reduction varied significantly. However, even in countries with strong surveillance systems, not all cases will be detected and modelling frameworks suggest that a substantial number of undiagnosed cases should be expected, resulting in residual reservoir transmission potential [19].

Possible other factors explaining inter‐country discrepancies include the timing of the influenza season relative to the introduction of NPIs in early 2020. In regions where the annual influenza epidemic was ongoing at NPI introduction [20], NPIs may have had limited impact on that year's seasonal wave. In countries with different influenza seasonality like New Zealand, NPIs have preceded widespread community transmission (typical influenza peak in New Zealand is around July/August) and thus had a greater effect in 2020 [21]. Naturally, in the latter case, the geography of New Zealand as an island nation simplifies circulation control across borders compared to a land nation with open borders.

Another factor impacting circulation patterns in different countries is the nature, consistency, and strictness of NPIs imposed, which are summarized by the COVID‐19 stringency index, ranging from 0 (least stringent) to 100 (most stringent) [22]. Countries maintaining stricter and longer‐lasting NPIs generally appeared to show greater reductions in influenza circulation. This relationship could not be formally analyzed because most included studies reported aggregated incidence data across broad time periods rather than detailed time‐specific estimates linked to changing NPI measures. For example, the USA had a moderate level of stringency around 70 throughout 2020, whereas other countries like Croatia had a high stringency index only in the first months of the pandemic (> 90 in April 2020) followed by relaxed NPIs with values < 50 in the second half of 2020, potentially contributing to the observed increase in influenza activity [22].

Interestingly, reductions in influenza were similar between the Northern and Southern Hemispheres, suggesting that contextual factors such as timing and stringency of NPIs played a more important role. Countries with stronger public health systems and more robust surveillance tended to show larger reductions, likely reflecting higher capacity for implementing NPIs effectively. Additionally, qualitative research has identified social factors like poverty, household structure, or informal workplaces to have posed major barriers in a population's capacity to adhere to NPIs [23]. Overall, local influenza patterns appear to result from a complex interplay of social, structural, and policy factors rather than economic status alone.

The largest descriptive reduction in influenza incidence was observed in the working‐age population (−80%), perhaps reflecting the effect of NPIs on high‐intensity contact spaces like in workplaces and public settings [24]. In contrast, smaller reductions appeared to occur in children more likely to be cared for at home (< 6 years: −66%), suggesting differences in the association between NPIs and influenza incidence across age groups. Older adults (≥ 65 years) also showed a more moderate reduction in influenza incidence (−62%). However, this estimate was based on a limited number of studies (*n* = 5) and should therefore be interpreted cautiously. Greater adherence to NPIs and prioritization in early influenza vaccination campaigns among older adults may have contributed to the observed reduction in this high‐risk age group [25, 26, 27].

This similarity of influenza incidence in hospital inpatients and outpatients likely represents a “tip of the iceberg” phenomenon where an overall reduction in community transmission results in fewer severe cases requiring hospitalization, as also conceptualized in other studies [19]. It may also be a function of the effectiveness of influenza vaccination campaigns in high‐risk populations with greater risk for hospitalization [25, 26]. Additionally, a focus on SARS‐CoV‐2 testing early in the pandemic may have led to under‐recognition or under‐reporting of outpatient influenza by reduced numbers of samples tested in certain settings, as seen e.g., in India or Argentina [28].

Our limited analysis on studies examining influenza strains and the year‐by‐year comparison reflect the near disappearance of the B Yamagata strain, consistent with recent reports suggesting the possible extinction or prolonged absence of the B/Yamagata lineage [29]. Continued surveillance needs to confirm longer‐term trends in influenza strain ecology and will enable e.g., changes in influenza vaccine composition and the theoretical risk of reintroduction via live vaccines or laboratory spillover events [30]. The temporal analysis globally revealed that the highest reduction in influenza incidence occurred in 2021, when NPIs were implemented most broadly and stringently across countries. By 2022, the observed reduction in influenza was reverted, likely reflecting the gradual relaxation of NPIs [22].

## 5 Limitations and Strengths

Firstly, the quality assessment indicates that most studies were methodologically sound, with clearly defined objectives, adequate and representative samples, and prespecified outcome measures. Incomplete reporting of recruitment procedures, testing conditions, and stratification limit the ability to fully assess bias and heterogeneity, yet this is partly expected because about half of the studies relied on surveillance databases that do not routinely provide such details. Nevertheless, the use of multi‐year data strengthens confidence in the findings and provides a solid foundation for interpreting trends.

Moreover, the inclusion of data from 115 articles spanning 98 countries enabled a robust and comprehensive synthesis of global evidence. However, the geographic distribution of included studies was disproportionately influenced by few countries, most notably China, Japan, and the USA, which may introduce publication or regional bias and limit the generalizability of findings to other settings. During the initial phase of the pandemic, NPIs were relatively similar for most settings with international travel restrictions. Later, policy heterogeneity presents an additional challenge [31] for interpretation: for instance, some countries like China implemented prolonged and stringent NPIs [32], while the USA exhibited greater variability over time and in different states [33].

Thirdly, several factors may have influenced the observed reduction in influenza cases, but these could not be accounted for within the scope of our analysis. For instance, vaccination policies, coverage rates, and their heterogeneous implementation across countries and time periods may affect influenza incidence independently of NPIs. However, vaccination coverage data were inconsistently reported across included studies and could not be standardized sufficiently to permit stratified analyses or adjustment in the meta‐analysis. Viral interference might also have contributed during the COVID‐19 pandemic, potentially introducing bias into the observed reduction attributed to NPIs. Furthermore, because our analysis is based on incidence counts, reductions in laboratory capacity resulting from the prioritization of COVID‐19 testing may have affected the reported numbers.

In addition to analyzing the overall pre‐ and post‐NPI periods, single‐year data were examined to provide insights into temporal changes in effects. However, data points were limited in the later phases of the study period, in particular in 2022, which constrained the ability to draw definitive temporal conclusions. This limitation may also reflect the increasing heterogeneity in NPI stringency across different regions worldwide.

Although the literature search was conducted in August 2023 and included studies published after the WHO declaration ending the COVID‐19 Public Health Emergency in May 2023 [34], the epidemiological data reported in the included studies extended only until April 2023. Thus, the effective temporal coverage of this review was determined by the availability of primary surveillance data rather than by an a priori eligibility criterion. As a result, the findings primarily reflect influenza dynamics during periods of active or recently relaxed NPIs and may not fully capture longer‐term post‐pandemic influenza circulation patterns.

Lastly, although stratification of our data enabled exploration of differential effects of certain variables on influenza incidence, the high level of data aggregation in many studies limits the robustness of these conclusions. Formal statistical comparisons between subgroups were not performed due to the limited number of studies within several strata and the aggregated nature of the available data. Therefore, observed differences between subgroups should be considered exploratory and should not be interpreted as evidence of statistically significant differences. Some substrata data may be incomplete or not fully generalizable. Furthermore, the aggregated nature of the data is too coarse to reliably correlate influenza trends with the timing of NPI implementation or relaxation. Consequently, this review cannot provide differential estimates for the impact of individual NPI measures.

Yet, despite its limitations, this review provides a comprehensive and in‐depth overview of the global impact of the COVID‐19 pandemic on influenza occurrence. Drawing on data from a large number of studies across diverse geographic regions, we present a robust and wide‐ranging synthesis of influenza dynamics during this period.

## 6 Conclusions

This systematic review quantifies the global impact of COVID‐19 on influenza incidence and informs efforts, such as the RESPINOW modeling consortium, to simulate respiratory infection transmission. We found an overall 92% reduction in influenza during the NPI period compared with pre‐pandemic seasons, with variations across regions, age groups, and patient populations, reflecting differences in NPI timing, stringency, prior influenza activity, and adherence. The largest reductions were descriptively observed in higher‐income countries, particularly in the WHO transmission zones of Northern Europe and Eastern Asia, and among high‐risk populations such as hospital inpatients and young children.

These results show that COVID‐19 NPIs had broad collateral effects on influenza transmission. Data limitations and aggregation highlight the need to strengthen global surveillance. Integrating detailed surveillance data into modeling initiatives like RESPINOW can improve understanding of NPI effects, guide targeted public health strategies, and enhance preparedness for future pandemics.

## Author Contributions

**Laura‐Inés Böhler:** data curation, writing – original draft, investigation, validation, supervision. **Lisa Koeppel:** methodology, data curation, formal analysis, writing – original draft, writing – review and editing, visualization, conceptualization, validation. **Stefan Fabian Weber:** validation, writing – original draft, writing – review and editing. **Mary Gaeddert:** writing – review and editing, methodology. **Katharina Thielemann:** data curation. **Kerstin Glaser:** data curation. **Horeya M. Ismail:** data curation. **Ulrich Reinacher:** formal analysis, methodology, writing – review and editing. **Veronika K. Jaeger:** data curation, writing – review and editing. **Julia Böhnke:** data curation, writing – review and editing. **Antonia Bartz:** writing – review and editing, data curation. **Maja Pavic:** data curation, writing – review and editing. **Manuela Harries:** data curation, writing – review and editing. **Christina Kuczewski:** data curation. **Torben Heinsohn:** data curation. **Olga Hovardovska:** data curation. **Sin‐Yin Huei:** data curation. **Chao Xu:** data curation, writing – review and editing. **Cornelia Gottschick:** data curation, writing – review and editing. **Seba Contreras:** data curation, writing – review and editing. **Maciej Filinski:** visualization. **Maurizio Grilli:** investigation. **Berit Lange:** supervision, funding acquisition. **Claudia M. Denkinger:** supervision, funding acquisition, writing – review and editing.

## Funding

This study was supported by the Federal Ministry of Education and Research (BMBF) via the RESPINOW (grant number: UKHD 031L0298C, HZI MV2021‐012, NMI FKZ031L0298B, MPI 031L0298G, University of Münster 031L0298F).

## Conflicts of Interest

The authors declare no conflicts of interest.

## Supporting information


**Data S1:** Supporting information.

**Table S1:** Supporting information.

## Data Availability

The data that support the findings of this study are available in the Supporting Information of this article.
