# Supplementary material for: Impact of Non‐Pharmaceutical Interventions Targeted at COVID‐19 Pandemic on Influenza Burden—A Systematic Review and Meta‐Analysis
Source: Influenza Other Respir Viruses. 2026 Jul 29;20(8):e70301. doi: 10.1111/irv.70301 (PMC13416394; doi:10.1111/irv.70301)
Supplement: Supplementary file 1 — Data S1: Supporting information. [file IRV-20-e70301-s001.docx]

**Supplementary Material to**

***Impact of non-pharmaceutical interventions targeted at COVID-19 pandemic on influenza burden—a systematic review and meta-analysis***

**S1: RESPINOW Study group members**

Alex Dulovic, Alex Kuhlmann, André Karch, Berit Lange, Carolina J. Klett-Tammen, Chao Xu, Claudia Denkinger, Cornelia Gottschick, Daniel Wolffram, Isti Rodiah, Johannes Bracher, Laura-Inés Boehler, Lisa Koeppel, Manuela Harries, Melanie Schienle, Michael Böhm, Nicole Schneiderhan-Marra, Nils Bardeck, Olga Hovardovska, Patrick Marsall, Philipp Dönges, Rafael Mikolajczyk, Rolf Kaiser, Sebastian Contreras, Torben Heinsohn, Tyll Krüger, Ulrich Reinacher, Veronika K. Jaeger, Viola Priesemann, Wolfgang Bock

**S2: List of Search terms**

## **PubMed**

| "Respiratory Syncytial Viruses"[Mesh] OR  "Respiratory Syncytial Virus Infections"[Mesh] OR  "Respiratory Syncytial"[tiab] OR  RSV[tiab] OR  RSVs[tiab] OR |
| --- |
| "Influenza, Human"[Mesh] OR  "Bronchiolitis"[Mesh] OR  Bronchiolit*[tiab] OR  Influenza*[tiab] OR  "Human Flu"[tiab] OR |
| "Streptococcus pneumoniae"[Mesh] OR  Pneumococcus[tiab] OR  "Diplococcus pneumoniae"[tiab] OR  "Streptococcus pneumoniae"[tiab] |

### AND

| "COVID-19"[Mesh] OR  "Coronavirus"[Mesh] OR  covid*[tw] OR  coronavirus*[tw] OR  corona virus*[tw] OR  ncov*[tw] OR  "n cov*"[tw] OR  sarscov*[tw] OR  "sars cov*"[tw] OR  2019nCoV*[tw] OR  "2019 nCoV*"[tw] OR  sars2*[tw] OR  "sars 2*"[tw] |
| --- |

## **Cochrane Library**

| [mh "Respiratory Syncytial Viruses"] OR  [mh "Respiratory Syncytial Virus Infections"] OR  (Respiratory NEAR Syncytial):ti,ab,kw OR  (RSV):ti,ab,kw OR  (RSVs):ti,ab,kw OR  [mh "Influenza, Human"] OR  [mh "Bronchiolitis"] OR  (Bronchiolit*):ti,ab,kw OR  (Influenza*):ti,ab,kw OR  (Human NEAR Flu):ti,ab,kw OR  [mh "Streptococcus pneumoniae"] OR  (Pneumococcus):ti,ab,kw OR  (Diplococcus NEAR pneumoniae):ti,ab,kw OR  (Streptococcus NEAR pneumoniae):ti,ab,kw |
| --- |

### AND

| [mh "COVID-19"] OR  [mh "Coronavirus"] OR  (covid*):ti,ab,kw OR  (coronavirus*):ti,ab,kw OR  (corona NEAR virus*):ti,ab,kw OR  (ncov*):ti,ab,kw OR  (n NEAR cov*):ti,ab,kw OR  (sarscov*):ti,ab,kw OR  (sars NEAR cov*):ti,ab,kw OR  (2019nCoV*):ti,ab,kw OR  (2019 NEAR nCoV*):ti,ab,kw OR  (sars2*):ti,ab,kw OR  (sars NEAR 2*):ti,ab,kw |
| --- |

## **Web of Science Core Collection**

TI=("Respiratory Syncytial" OR "RSV" OR "RSVs" OR "Bronchiolit*" OR "Influenza*" OR "Human Flu" OR "Pneumococcus" OR "Diplococcus pneumoniae" OR "Streptococcus pneumoniae" ) OR AB=("Respiratory Syncytial" OR "RSV" OR "RSVs" OR "Bronchiolit*" OR "Influenza*" OR "Human Flu" OR "Pneumococcus" OR "Diplococcus pneumoniae" OR "Streptococcus pneumoniae" )

AND

TI=("covid*" OR "coronavirus*" OR "corona virus*" OR "ncov*" OR "n cov*" OR "sarscov*" OR "sars cov*" OR "2019nCoV*" OR "2019 nCoV*" OR "sars2*" OR "sars 2*") OR AB=("covid*" OR "coronavirus*" OR "corona virus*" OR "ncov*" OR "n cov*" OR "sarscov*" OR "sars cov*" OR "2019nCoV*" OR "2019 nCoV*" OR "sars2*" OR "sars 2*")

##

## **MedRxiv BioRxiv (über Europe PMC)**

("Respiratory Syncytial" OR "RSV" OR "RSVs" OR "Bronchiolit*" OR "Influenza*" OR "Human Flu" OR "Pneumococcus" OR "Diplococcus pneumoniae" OR "Streptococcus pneumoniae")

AND

("covid*" OR "coronavirus*" OR "corona virus*" OR "ncov*" OR "n cov*" OR "sarscov*" OR "sars cov*" OR "2019nCoV*" OR "2019 nCoV*" OR "sars2*" OR "sars 2*")

AND

(PUBLISHER:MedRxiv OR PUBLISHER:BioRxiv)

**S3: PRISMA checklist**

| **Section and Topic** | **Item #** | **Checklist item** | **Location where item is reported** |
| --- | --- | --- | --- |
| **TITLE** | | |  |
| Title | 1 | Identify the report as a systematic review. | Title |
| **ABSTRACT** | | |  |
| Abstract | 2 | See the PRISMA 2020 for Abstracts checklist. | Abstract |
| **INTRODUCTION** | | |  |
| Rationale | 3 | Describe the rationale for the review in the context of existing knowledge. | Introduction, third and fourth paragraph |
| Objectives | 4 | Provide an explicit statement of the objective(s) or question(s) the review addresses. | Introduction, last paragraph |
| **METHODS** | | |  |
| Eligibility criteria | 5 | Specify the inclusion and exclusion criteria for the review and how studies were grouped for the syntheses. | Methods, Inclusion and exclusion |
| Information sources | 6 | Specify all databases, registers, websites, organisations, reference lists and other sources searched or consulted to identify studies. Specify the date when each source was last searched or consulted. | Methods, first paragraph |
| Search strategy | 7 | Present the full search strategies for all databases, registers and websites, including any filters and limits used. | Supplement, S2 |
| Selection process | 8 | Specify the methods used to decide whether a study met the inclusion criteria of the review, including how many reviewers screened each record and each report retrieved, whether they worked independently, and if applicable, details of automation tools used in the process. | Methods, Inclusion and exclusion; Screening and data extraction |
| Data collection process | 9 | Specify the methods used to collect data from reports, including how many reviewers collected data from each report, whether they worked independently, any processes for obtaining or confirming data from study investigators, and if applicable, details of automation tools used in the process. | Methods, Screening and data extraction |
| Data items | 10a | List and define all outcomes for which data were sought. Specify whether all results that were compatible with each outcome domain in each study were sought (e.g. for all measures, time points, analyses), and if not, the methods used to decide which results to collect. | Methods, Screening and data extraction |
|  | 10b | List and define all other variables for which data were sought (e.g. participant and intervention characteristics, funding sources). Describe any assumptions made about any missing or unclear information. | Methods, Screening and data extraction |
| Study risk of bias assessment | 11 | Specify the methods used to assess risk of bias in the included studies, including details of the tool(s) used, how many reviewers assessed each study and whether they worked independently, and if applicable, details of automation tools used in the process. | Methods, Risk of bias assessment |
| Effect measures | 12 | Specify for each outcome the effect measure(s) (e.g. risk ratio, mean difference) used in the synthesis or presentation of results. | Methods, Statistical analysis |
| Synthesis methods | 13a | Describe the processes used to decide which studies were eligible for each synthesis (e.g. tabulating the study intervention characteristics and comparing against the planned groups for each synthesis (item #5)). | Methods, Statistical analysis |
|  | 13b | Describe any methods required to prepare the data for presentation or synthesis, such as handling of missing summary statistics, or data conversions. | Methods, Statistical analysis |
|  | 13c | Describe any methods used to tabulate or visually display results of individual studies and syntheses. | Methods, Statistical analysis |
|  | 13d | Describe any methods used to synthesize results and provide a rationale for the choice(s). If meta-analysis was performed, describe the model(s), method(s) to identify the presence and extent of statistical heterogeneity, and software package(s) used. | Methods, Statistical analysis |
|  | 13e | Describe any methods used to explore possible causes of heterogeneity among study results (e.g. subgroup analysis, meta-regression). | Methods, Statistical analysis |
|  | 13f | Describe any sensitivity analyses conducted to assess robustness of the synthesized results. | Methods, Statistical analysis |
| Reporting bias assessment | 14 | Describe any methods used to assess risk of bias due to missing results in a synthesis (arising from reporting biases). | Methods, Risk of bias assessment |
| Certainty assessment | 15 | Describe any methods used to assess certainty (or confidence) in the body of evidence for an outcome. | Methods, Statistical analysis |
| **RESULTS** | | |  |
| Study selection | 16a | Describe the results of the search and selection process, from the number of records identified in the search to the number of studies included in the review, ideally using a flow diagram. | Results, Figure 1 |
|  | 16b | Cite studies that might appear to meet the inclusion criteria, but which were excluded, and explain why they were excluded. | Results, Figure 1 |
| Study characteristics | 17 | Cite each included study and present its characteristics. | Results, Figure 2; Supplement S5 |
| Risk of bias in studies | 18 | Present assessments of risk of bias for each included study. | Supplement S5 |
| Results of individual studies | 19 | For all outcomes, present, for each study: (a) summary statistics for each group (where appropriate) and (b) an effect estimate and its precision (e.g. confidence/credible interval), ideally using structured tables or plots. | Supplement S6 – S9; Results Figure 3 - 4 |
| Results of syntheses | 20a | For each synthesis, briefly summarise the characteristics and risk of bias among contributing studies. | Results |
|  | 20b | Present results of all statistical syntheses conducted. If meta-analysis was done, present for each the summary estimate and its precision (e.g. confidence/credible interval) and measures of statistical heterogeneity. If comparing groups, describe the direction of the effect. | Results, Supplement S6 – S9; Results Figure 3 - 4 |
|  | 20c | Present results of all investigations of possible causes of heterogeneity among study results. | Results |
|  | 20d | Present results of all sensitivity analyses conducted to assess the robustness of the synthesized results. | Results |
| Reporting biases | 21 | Present assessments of risk of bias due to missing results (arising from reporting biases) for each synthesis assessed. | Methods, Inclusion and exclusion |
| Certainty of evidence | 22 | Present assessments of certainty (or confidence) in the body of evidence for each outcome assessed. | Results, Supplement S6 – S9; Results Figure 3 - 4 |
| **DISCUSSION** | | |  |
| Discussion | 23a | Provide a general interpretation of the results in the context of other evidence. | Discussion |
|  | 23b | Discuss any limitations of the evidence included in the review. | Discussion |
|  | 23c | Discuss any limitations of the review processes used. | Discussion |
|  | 23d | Discuss implications of the results for practice, policy, and future research. | Discussion |
| **OTHER INFORMATION** | | |  |
| Registration and protocol | 24a | Provide registration information for the review, including register name and registration number, or state that the review was not registered. | Methods, first paragraph |
|  | 24b | Indicate where the review protocol can be accessed, or state that a protocol was not prepared. | Methods, first paragraph |
|  | 24c | Describe and explain any amendments to information provided at registration or in the protocol. | / |
| Support | 25 | Describe sources of financial or non-financial support for the review, and the role of the funders or sponsors in the review. | Funding |
| Competing interests | 26 | Declare any competing interests of review authors. | Conflict of interest |
| Availability of data, code and other materials | 27 | Report which of the following are publicly available and where they can be found: template data collection forms; data extracted from included studies; data used for all analyses; analytic code; any other materials used in the review. | Supplement |

*This checklist is taken from* Page MJ, McKenzie JE, Bossuyt PM, Boutron I, Hoffmann TC, Mulrow CD, et al. The PRISMA 2020 statement: an updated guideline for reporting systematic reviews. BMJ 2021;372:n71. doi: 10.1136/bmj.n71. This work is licensed under CC BY 4.0. To view a copy of this license, visit https://creativecommons.org/licenses/by/4.0/

**S4: Modified NHLBI Quality assessment**

| Signaling Question (Responses were Yes, No, Unclear) |
| --- |
| 1. Was the study question or objective clearly stated? |
| 1. Were eligibility/selection criteria for the study population prespecified and clearly described? |
| 1. Were the participants in the study representative of those who would be in the general or clinical population of interest? |
| 1. Was the sample size sufficiently large to provide confidence in the findings? |
| 1. Was the testing for disease clearly described and delivered consistently across the study population? |
| 1. Were the outcome measures prespecified, clearly defined, valid, reliable, and assessed consistently across all study participants? |
| 1. Were outcome measures of interest taken multiple years/seasons before the COVID-19 pandemic and multiple years/seasons during the pandemic? |
| 1. Did the study consider strata (e.g. age groups or different hospitals) in the statistical analysis to determine differences in subgroups? |

**S5: NHLBI Quality assessment results by paper**

| **Acronym** | **Objective** | **Recruitment** | **Representative** | **Sample Size** | **Testing** | **Outcome** | **Multi-year** | **Stratification** |
| --- | --- | --- | --- | --- | --- | --- | --- | --- |
| Abo 2021 ^1^ | Yes | Yes | Yes | Yes | Yes | Yes | Yes | Yes |
| Adegbija 2021 ^2^ | Yes | Yes | Yes | Yes | No | Yes | Yes | No |
| Akhtar 2021 ^3^ | Yes | Yes | Yes | Yes | Yes | Yes | Yes | No |
| Alaib 2023 ^4^ | Yes | Yes | Yes | Yes | Yes | Yes | Yes | Yes |
| Avolio 2022 ^5^ | Yes | No | Unclear | Yes | Yes | Yes | Yes | No |
| Berdah 2022 ^6^ | Yes | Yes | Yes | Yes | Yes | Yes | Yes | Yes |
| Bhardwaj 2022 ^7^ | Yes | Yes | Yes | Yes | Yes | Yes | Yes | Yes |
| Bhatt 2022 ^8^ | Yes | Yes | Yes | Yes | Yes | Yes | Yes | No |
| Bögli 2023 ^9^ | Yes | Yes | Yes | Yes | Yes | Yes | Yes | No |
| Bowyer 2022 ^10^ | Yes | Yes | Yes | Yes | No | Unclear | Yes | No |
| Brehm 2022 ^11^ | Yes | No | Yes | Yes | No | Yes | Yes | Yes |
| Bright 2020 ^12^ | Yes | Yes | Yes | Yes | Yes | Yes | Yes | No |
| Burks 2023 ^13^ | Yes | Yes | Yes | Yes | No | Yes | Yes | Yes |
| Cao 2023 ^14^ | Yes | Yes | Yes | Yes | Yes | Yes | Yes | Yes |
| Cao 2023 ^15^ | Yes | Yes | Yes | Yes | Yes | Yes | Yes | Yes |
| Cardenas 2022 ^16^ | Yes | Yes | Yes | No | No | Yes | No | Yes |
| Chen 2021 ^17^ | Yes | No | Yes | Yes | Unclear | Yes | No | No |
| Chiu 2020 ^18^ | Yes | No | Yes | Yes | Yes | Yes | Yes | Yes |
| CiofiDegliAtti 2023 ^19^ | Yes | Yes | No | Yes | Yes | Yes | Yes | Yes |
| Clinciu 2021 ^20^ | Unclear | No | Yes | Yes | No | Yes | No | No |
| Cui 2022 ^21^ | Yes | Yes | Yes | Yes | Yes | Yes | Yes | Yes |
| Davis 2022 ^22^ | Yes | No | Yes | Yes | No | Yes | Yes | Yes |
| Deleveaux 2022 ^23^ | No | No | Yes | Yes | No | Unclear | Yes | Yes |
| DeRose 2022 ^24^ | Yes | Yes | No | No | Yes | Yes | Yes | No |
| Dhanasekaran 2022 ^25^ | Yes | No | Yes | Yes | No | Yes | Yes | Yes |
| Dong 2021 ^26^ | No | No | Yes | Yes | No | No | Yes | No |
| Doroshenko 2021 ^27^ | Yes | No | Yes | Yes | Yes | Yes | No | No |
| Du 2021 ^28^ | Yes | Yes | Yes | Yes | Yes | Yes | Yes | Yes |
| Feng 2023 ^29^ | Yes | Yes | Yes | No | Yes | Yes | No | Yes |
| Fukuda 2021 ^30^ | Yes | Yes | No | No | No | Yes | No | Yes |
| Fukuda 2023 ^31^ | Yes | Yes | No | Yes | No | Yes | No | Yes |
| García-García 2022 ^32^ | Yes | Yes | No | Yes | Yes | Yes | No | No |
| George 2022 ^33^ | No | No | Unclear | Yes | No | Unclear | Yes | No |
| Gilca 2022 ^34^ | Yes | Yes | Yes | Yes | No | Yes | No | Yes |
| Giraud-Gatineau 2022 ^35^ | Yes | Yes | Yes | Yes | Yes | Yes | Yes | Yes |
| Groves 2021 ^36^ | Yes | No | Yes | Yes | No | Yes | Yes | Yes |
| Guo 2022 ^37^ | Yes | No | Yes | Yes | Yes | Yes | Yes | No |
| Habbous 2023 ^38^ | Yes | No | Yes | Yes | No | Yes | Yes | Yes |
| Haddadin 2022 ^39^ | Yes | Yes | Yes | Yes | Yes | Yes | Yes | Yes |
| Heinzinger 2021 ^40^ | Yes | Yes | Yes | Yes | Yes | Yes | No | Yes |
| Hirae 2023 ^41^ | Yes | No | Yes | Yes | Unclear | Yes | Yes | Yes |
| Hsu 2021 ^42^ | Yes | No | No | Yes | Yes | Yes | Yes | No |
| Hu 2021 ^43^ | Yes | No | Yes | Yes | No | Yes | Yes | No |
| Hu 2022 ^44^ | Yes | No | Yes | Yes | No | Yes | Yes | Yes |
| Huang 2022 ^45^ | Yes | No | Yes | Yes | No | Yes | Yes | Yes |
| Hwang 2023 ^46^ | Yes | Yes | Yes | Yes | No | Yes | Yes | Yes |
| Ippolito 2021 ^47^ | Yes | Yes | No | No | Yes | Yes | Yes | Yes |
| Izu 2023 ^48^ | Yes | Yes | Yes | Yes | No | Yes | Yes | Yes |
| Jayaram 2022 ^49^ | Yes | Yes | Yes | Yes | Yes | Yes | Yes | Yes |
| Kadambari 2022 ^50^ | Yes | No | Yes | Yes | Unclear | Yes | Yes | No |
| Kanda 2023 ^51^ | Yes | No | Yes | Yes | No | Yes | Yes | No |
| Kim 2022 ^52^ | Yes | Yes | Yes | Yes | Yes | Yes | Yes | No |
| Knudsen 2022 ^53^ | Yes | Yes | Yes | Yes | No | Yes | Yes | No |
| Kume 2022 ^54^ | Yes | Yes | Yes | Yes | No | Yes | Yes | Yes |
| Lampros 2023 ^55^ | Yes | Yes | Yes | Yes | Yes | Yes | Yes | Yes |
| LamraniHanchi 2022 ^56^ | Yes | Yes | Yes | No | Yes | Yes | Yes | Yes |
| Lee 2021 ^57^ | Yes | No | Yes | Yes | No | Yes | Yes | Yes |
| Leng 2021 ^58^ | Yes | Yes | Yes | Yes | Yes | Yes | No | Yes |
| Li 2021 ^59^ | Yes | Yes | Yes | Yes | Yes | Yes | No | No |
| Li 2022 ^60^ | Yes | Yes | Yes | No | Yes | Yes | Yes | Yes |
| Liu 2021 ^61^ | Yes | Yes | Yes | Yes | Yes | Yes | No | Yes |
| Liu 2022 ^62^ | Yes | Yes | Yes | Yes | Yes | Yes | Yes | Yes |
| Liu 2023 ^63^ | Yes | Yes | Yes | No | Yes | Yes | No | Yes |
| Liu 2023 ^64^ | Yes | Yes | Yes | Yes | Yes | Yes | Yes | Yes |
| Lu 2022 ^65^ | Yes | Yes | Yes | Yes | Yes | Yes | Yes | Yes |
| Lumley 2022 ^66^ | Yes | No | Yes | Yes | Yes | Yes | Yes | Yes |
| Łuniewska 2023 ^67^ | Yes | No | Yes | Yes | No | Yes | Yes | Yes |
| Luo 2020 ^68^ | Unclear | No | Yes | No | No | No | No | No |
| Maison 2022 ^69^ | No | Yes | Yes | Yes | Yes | Yes | Yes | Yes |
| MárquezCaballero 2023 ^70^ | Yes | No | Yes | No | No | No | Yes | No |
| Maruo 2022 ^71^ | Yes | No | Yes | Yes | Yes | Yes | Yes | No |
| Moscovich 2023 ^72^ | Yes | No | Yes | No | No | Yes | Yes | No |
| Nott 2022 ^73^ | Yes | No | Yes | Yes | No | No | Yes | Yes |
| Nwosu 2021 ^74^ | Yes | No | Yes | Yes | No | Yes | No | No |
| Park 2021 ^75^ | Yes | No | Yes | Yes | No | Yes | Yes | No |
| Parry 2020 ^76^ | Yes | No | Yes | No | Yes | Yes | Yes | No |
| Perez 2022 ^77^ | No | Yes | Yes | Yes | No | Yes | Yes | No |
| Pierce 2021 ^78^ | Yes | No | Yes | Yes | No | Yes | Yes | Yes |
| Polcwiartek 2021 ^79^ | Yes | Yes | Yes | Yes | No | No | Yes | No |
| Razanajatovo 2022 ^80^ | Yes | Yes | Yes | Yes | Yes | Yes | Yes | Yes |
| Ren 2023 ^81^ | Yes | Yes | Yes | Yes | Yes | Yes | No | No |
| Sachs 2023 ^82^ | Yes | Yes | No | Yes | Yes | Yes | Yes | No |
| Sakamoto 2020 ^83^ | Yes | No | Yes | Yes | No | Yes | Yes | No |
| Sberna 2022 ^84^ | Yes | Yes | Yes | No | Yes | Yes | Yes | No |
| Serigstad 2022 ^85^ | Yes | Yes | Yes | No | Yes | Yes | Yes | No |
| Shi 2022 ^86^ | Yes | Yes | Yes | No | Yes | Yes | Yes | No |
| Shi 2023 ^87^ | Yes | No | Yes | Yes | No | Yes | Yes | No |
| Shichijo 2021 ^88^ | Yes | Yes | No | Yes | Unclear | Yes | Yes | No |
| Song 2022 ^89^ | Yes | No | Yes | Yes | No | Yes | Yes | Yes |
| Stamm 2021 ^90^ | Yes | Yes | Yes | Yes | Yes | Yes | Yes | Yes |
| Steinfort 2020 ^91^ | No | No | Yes | Yes | No | No | Yes | No |
| Sullivan 2020 ^92^ | No | No | Yes | Yes | No | No | Yes | No |
| Suntronwong 2020 ^93^ | No | No | Unclear | Yes | Unclear | Unclear | No | No |
| Tan 2023 ^94^ | Yes | Yes | Yes | Yes | Yes | Yes | Yes | Yes |
| Tang 2022 ^95^ | Yes | No | Yes | Yes | No | Yes | Yes | No |
| Teutsch 2021 ^96^ | Yes | Yes | Yes | No | Yes | Yes | Yes | Yes |
| Trenholme 2021 ^97^ | Unclear | Unclear | No | No | Unclear | Yes | Yes | Yes |
| Uhteg 2022 ^98^ | Yes | Yes | Yes | No | Yes | Yes | No | Yes |
| Venkatram 2022 ^99^ | Yes | Yes | Yes | Yes | Yes | Yes | Yes | Yes |
| Vila 2023 ^100^ | Yes | Yes | Yes | Yes | No | Yes | Yes | Yes |
| Vittucci 2021 ^101^ | Yes | Yes | No | Yes | Yes | Yes | Yes | No |
| Wagatsuma 2022 ^102^ | Yes | Yes | Yes | Yes | Yes | Yes | Yes | No |
| Wang 2021 ^103^ | Yes | Yes | No | Yes | Yes | Yes | Yes | No |
| Wang 2023 ^104^ | Yes | Yes | Yes | Yes | Yes | Yes | Yes | Yes |
| Wu 2022 ^105^ | Yes | No | Yes | Yes | No | Yes | Yes | Yes |
| Xiao 2021 ^106^ | Yes | No | Yes | Yes | No | Yes | Yes | No |
| Xu 2022 ^107^ | Yes | Yes | Yes | Yes | Yes | Yes | No | Yes |
| Ye 2022 ^108^ | Yes | Yes | Yes | Yes | Yes | Yes | No | Yes |
| Ye 2023 ^109^ | Yes | Yes | Yes | Yes | Yes | Yes | Yes | Yes |
| Yeoh 2021 ^110^ | Yes | No | Yes | Yes | No | Yes | Yes | No |
| Yi 2023 ^111^ | Yes | Yes | Yes | Yes | Yes | Yes | Yes | Yes |
| Yorsaeng 2022 ^112^ | Yes | No | Yes | Yes | No | Yes | No | No |
| Zhang 2023 ^113^ | Yes | No | Yes | Yes | No | Yes | No | Yes |
| Zheng 2022 ^114^ | Yes | Yes | Yes | Yes | Yes | Yes | Yes | Yes |
| Zuo 2023 ^115^ | Yes | No | Yes | Yes | No | Yes | Yes | No |

**S6: Forest plot of the relative change in influenza incidence for the full pandemic period compared to the pre-pandemic period, stratified by Influenza transmission zones. The dot size denotes the number of data-reporting papers contributing to each stratum. An open circle indicates that data were reported by a single paper.**


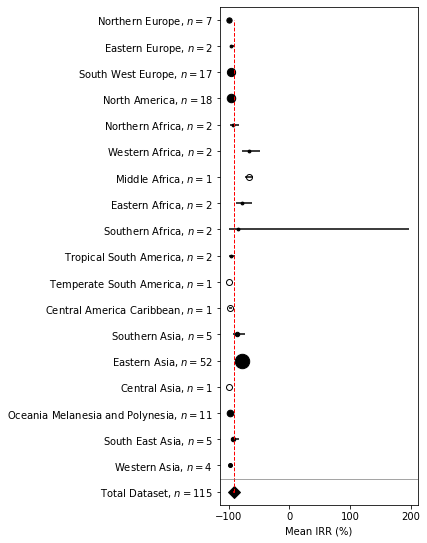


**S7: Forest plot of the relative change in influenza incidence for the full pandemic period compared to the pre-pandemic period, stratified by WTO classification. The dot size denotes the number of data-reporting papers contributing to each stratum.**


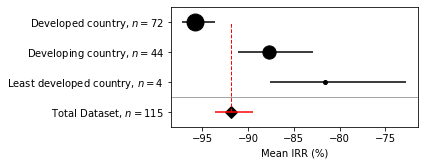


**S8: Forest plot of the relative change in influenza incidence for the full pandemic period compared to the pre-pandemic period, stratified by Human Development Index (HDI) classification. The dot size denotes the number of data-reporting papers contributing to each stratum.**


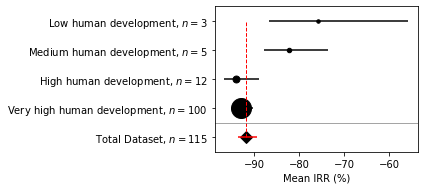


**S9: Forest plot of the relative change in influenza incidence for the full pandemic period compared to the pre-pandemic period, stratified by strain and substrain. The dot size denotes the number of data-reporting papers contributing to each stratum. All influenza A substrains are included in the influenza A strain, analogously for influenza B strains. Therefore, categories are not mutually exclusive, and some data may contribute to multiple strata. The “Total Dataset” additionally includes studies that did not report strain information or provided data for all strains combined.**


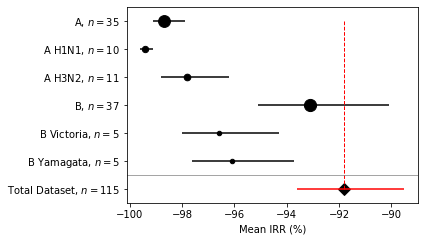


**S10:** **Spatial distribution of relative change in influenza incidence from the pre-pandemic period to A) the full pandemic period B) only 2020, C) only 2021, and D) only 2022. The white color denotes missing data.**


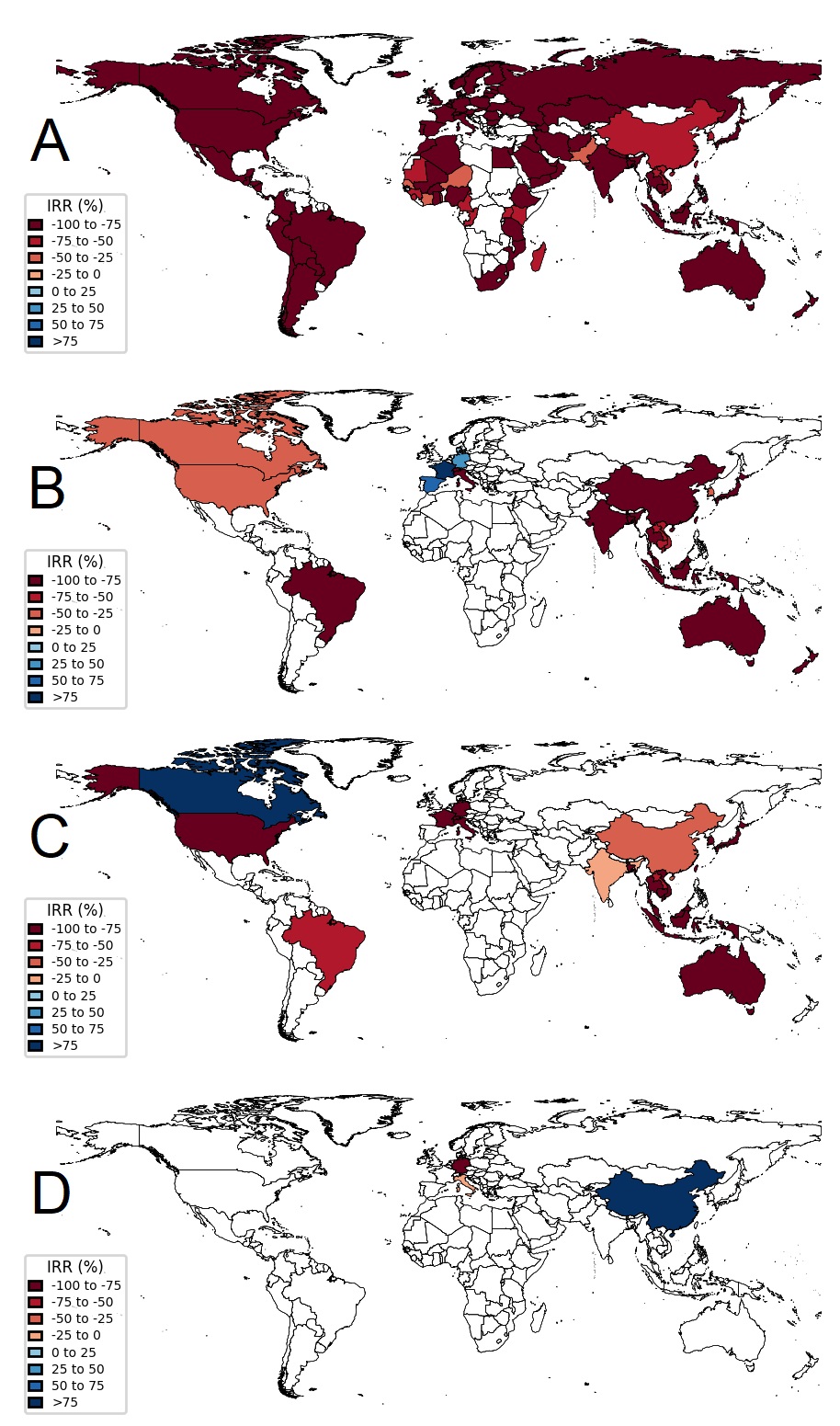


**References**

**List of references from papers included in the systematic review**

1. Abo YN, Clifford V, Lee LY, et al. COVID-19 public health measures and respiratory viruses in children in Melbourne. *Journal of Paediatrics and Child Health*. 2021;doi:10.1111/jpc.15601

2. Adegbija O, Walker J, Smoll N, Khan A, Graham J, Khandaker G. Notifiable diseases after implementation of COVID-19 public health prevention measures in Central Queensland, Australia. *Commun Dis Intell (2018)*. 2021;45doi:10.33321/cdi.2021.45.11

3. Akhtar Z, Chowdhury F, Rahman M, et al. Seasonal influenza during the COVID-19 pandemic in Bangladesh. *PLoS One*. 2021;16(8):e0255646. doi:10.1371/journal.pone.0255646

4. Alaib H, Algariri N, Ahmed H, et al. Frequency and Seasonal Variations of Viruses Causing Respiratory Tract Infections in Children Pre- and Post-COVID-19 Pandemic in Riyadh (2017-2022). *Cureus*. 2023;15(1):e33467. doi:10.7759/cureus.33467

5. Avolio M, Venturini S, De Rosa R, Crapis M, Basaglia G. Epidemiology of respiratory virus before and during COVID-19 pandemic. *Infez Med*. 2022;30(1):104-108. doi:10.53854/liim-3001-12

6. Berdah L, Romain AS, Rivière S, et al. Retrospective observational study of the influence of the COVID-19 outbreak on infants' hospitalisation for acute bronchiolitis. *BMJ Open*. 2022;12(10):e059626. doi:10.1136/bmjopen-2021-059626

7. Bhardwaj S, Choudhary ML, Jadhav S, et al. A retrospective analysis of respiratory virus transmission before and during the COVID-19 pandemic in Pune the western region of India. *Front Public Health*. 2022;10:936634. doi:10.3389/fpubh.2022.936634

8. Bhatt P, Strachan J, Easton M, Franklin L, Drewett G. Effect of COVID-19 restrictions and border closures on vaccine preventable diseases in Victoria, Australia, 2020-2021. *Commun Dis Intell (2018)*. 2022;46. doi:10.33321/cdi.2022.46.29

9. Bögli J, Güsewell S, Strässle R, Kahlert CR, Albrich WC. Pediatric hospital admissions, case severity, and length of hospital stay during the first 18 months of the COVID-19 pandemic in a tertiary children's hospital in Switzerland. *Infection*. 2023;51(2):439-446. doi:10.1007/s15010-022-01911-x

10. Bowyer SA, Bryant WA, Key D, et al. Machine learning forecasting for COVID-19 pandemic-associated effects on paediatric respiratory infections. *Arch Dis Child*. 2022;doi:10.1136/archdischild-2022-323822

11. Brehm TT, Hennigs A. [Seasonal Influenza - Update on Epidemiology, Prevention and Therapy]. *Dtsch Med Wochenschr*. 2022;147(22):1456-1464. doi:10.1055/a-1838-9223

12. Bright A, Glynn-Robinson AJ, Kane S, Wright R, Saul N. The effect of COVID-19 public health measures on nationally notifiable diseases in Australia: preliminary analysis. *Commun Dis Intell (2018)*. 2020;44. doi:10.33321/cdi.2020.44.85

13. Burks A, King W, Orr M. The changing virology and trends in resource utilization for bronchiolitis since COVID-19. *Pediatr Pulmonol*. 2023;doi:10.1002/ppul.26640

14. Cao R, Du Y, Tong J, et al. Influence of COVID-19 pandemic on the virus spectrum in children with respiratory infection in Xuzhou, China: a long-term active surveillance study from 2015 to 2021. *BMC Infect Dis*. 2023;23(1):467. doi:10.1186/s12879-023-08247-3

15. Cao G, Guo Z, Liu J, Liu M. Change from low to out-of-season epidemics of influenza in China during the COVID-19 pandemic: A time series study. *J Med Virol*. 2023;95(6):e28888. doi:10.1002/jmv.28888

16. Cardenas J, Pringle C, Filipp SL, Gurka MJ, Ryan KA, Avery KL. Changes in Critical Bronchiolitis After COVID-19 Lockdown. *Cureus*. 2022;14(5):e25064. doi:10.7759/cureus.25064

17. Chen B, Wang M, Huang X, et al. Changes in Incidence of Notifiable Infectious Diseases in China Under the Prevention and Control Measures of COVID-19. *Front Public Health*. 2021;9:728768. doi:10.3389/fpubh.2021.728768

18. Chiu NC, Chi H, Tai YL, et al. Impact of Wearing Masks, Hand Hygiene, and Social Distancing on Influenza, Enterovirus, and All-Cause Pneumonia During the Coronavirus Pandemic: Retrospective National Epidemiological Surveillance Study. *J Med Internet Res*. 2020;22(8):e21257. doi:10.2196/21257

19. Ciofi Degli Atti M, Rizzo C, D'Amore C, et al. Acute respiratory infection emergency access in a tertiary care children hospital in Italy, prior and after the SARS-CoV-2 emergence. *Influenza Other Respir Viruses*. 2023;17(3):e13102. doi:10.1111/irv.13102

20. Clinciu DL, Chen CH, Wang JY, Huang G. Letter to the Editor: Impact of COVID-19 Prevention Methods on Influenza and Other Respiratory Infections in Taiwan. *Popul Health Manag*. 2021;24(5):631-632. doi:10.1089/pop.2021.0140

21. Cui A, Xie Z, Xu J, et al. Comparative analysis of the clinical and epidemiological characteristics of human influenza virus versus human respiratory syncytial virus versus human metapneumovirus infection in nine provinces of China during 2009-2021. *J Med Virol*. 2022;doi:10.1002/jmv.28073

22. Davis WW, Mott JA, Olsen SJ. The role of non-pharmaceutical interventions on influenza circulation during the COVID-19 pandemic in nine tropical Asian countries. *Influenza Other Respir Viruses*. 2022;16(3):568-576. doi:10.1111/irv.12953

23. Deleveaux S, Mekhaiel E. SEASONAL INFLUENZA IN THE WAKE OF THE NOVEL CORONAVIRUS PANDEMIC: AN EXAMPLE OF VIRAL INTERFERENCE? *Critical Care Medicine*. 2022;50(1):57-57. doi:10.1097/01.ccm.0000806912.17624.11

24. De Rose DU, Caoci S, Auriti C, et al. Lessons from SARS-CoV-2 Pandemics: How Restrictive Measures Impacted the Trend of Respiratory Infections in Neonates and Infants up to Three Months of Age. *Pathogens*. 2022;11(10). doi:10.3390/pathogens11101086

25. Dhanasekaran V, Sullivan S, Edwards KM, et al. Human seasonal influenza under COVID-19 and the potential consequences of influenza lineage elimination. *Nat Commun*. 2022;13(1):1721. doi:10.1038/s41467-022-29402-5

26. Dong M, Luo M, Li A, et al. Changes in the pathogenic spectrum of acute respiratory tract infections during the COVID-19 epidemic in Beijing, China: A large-scale active surveillance study. *Journal of Infection*. 2021;83(5):607-635. doi:10.1016/j.jinf.2021.08.013

27. Doroshenko A, Lee N, MacDonald C, Zelyas N, Asadi L, Kanji JN. Decline of Influenza and Respiratory Viruses With COVID-19 Public Health Measures: Alberta, Canada. *Mayo Clinic Proceedings*. 2021;96(12):3042-3052. doi:10.1016/j.mayocp.2021.09.004

28. Du X, Wu G, Zhu Y, Zhang S. Exploring the epidemiological changes of common respiratory viruses since the COVID-19 pandemic: a hospital study in Hangzhou, China. *Archives of Virology*. 2021;166(11):3085-3092. doi:10.1007/s00705-021-05214-8

29. Feng Z, Xu B, Zhong L, et al. A multicentre study on the incidence of respiratory viruses in children with community-acquired pneumonia requiring hospitalization in the setting of the zero-COVID policy in China. *Arch Virol*. 2023;168(2):64. doi:10.1007/s00705-023-05698-6

30. Fukuda Y, Tsugawa T, Nagaoka Y, et al. Surveillance in hospitalized children with infectious diseases in Japan: Pre- and post-coronavirus disease 2019. *Journal of Infection and Chemotherapy*. 2021;27(11):1639-1647. doi:10.1016/j.jiac.2021.07.024

31. Fukuda Y, Togashi A, Hirakawa S, et al. Changing Patterns of Infectious Diseases Among Hospitalized Children in Hokkaido, Japan, in the Post-COVID-19 Era, July 2019 to June 2022. *Pediatr Infect Dis J*. 2023;42(9):766-773. doi:10.1097/inf.0000000000003982

32. García-García E, Rodríguez-Pérez M, Melón García S, et al. Change on the Circulation of Respiratory Viruses and Pediatric Healthcare Utilization during the COVID-19 Pandemic in Asturias, Northern Spain. *Children (Basel)*. 2022;9(10). doi:10.3390/children9101464

33. George CR, Booy R, Nissen MD, Lahra MM. The decline of invasive meningococcal disease and influenza in the time of COVID-19: the silver linings of the pandemic playbook. *Med J Aust*. 2022;216(10):504-507. doi:10.5694/mja2.51463

34. Gilca R, Amini R, Carazo S, et al. The changing landscape of respiratory viruses contributing to respiratory hospitalisations: results from a hospital-based surveillance in Quebec, Canada, 2012-13 to 2021-22. medRxiv; 2022.

35. Giraud-Gatineau A, Kaba L, Boschi C, et al. Control of common viral epidemics but not of SARS-CoV-2 through the application of hygiene and distancing measures. *J Clin Virol*. 2022;150-151:105163. doi:10.1016/j.jcv.2022.105163

36. Groves HE, Piché-Renaud P, Peci A, et al. The impact of the COVID-19 pandemic on influenza, respiratory syncytial virus, and other seasonal respiratory virus circulation in Canada. medRxiv; 2021.

37. Guo MM, Yang KD, Liu SF, Kuo HC. Number of Kawasaki Disease Admissions Is Associated with Number of Domestic COVID-19 and Severe Enterovirus Case Numbers in Taiwan. *Children (Basel)*. 2022;9(2). doi:10.3390/children9020149

38. Habbous S, Hota S, Allen VG, Henry M, Hellsten E. Changes in hospitalizations and emergency department respiratory viral diagnosis trends before and during the COVID-19 pandemic in Ontario, Canada. *PLoS One*. 2023;18(6):e0287395. doi:10.1371/journal.pone.0287395

39. Haddadin Z, Spieker AJ, Rahman H, et al. Respiratory pathogens during the COVID-19 pandemic: Alterations in detection and seasonality in Nashville, Tennessee. *PLoS One*. 2022;17(8):e0270469. doi:10.1371/journal.pone.0270469

40. Heinzinger S, Eberle U, Angermeier H, et al. Reciprocal circulation pattern of SARS-CoV-2 and influenza viruses during the influenza seasons 2019/2020 and 2020/2021 in the Bavarian Influenza Sentinel (Germany). *Epidemiol Infect*. 2021;149:e226. doi:10.1017/s0950268821002296

41. Hirae K, Hoshina T, Koga H. Impact of the COVID-19 pandemic on the epidemiology of other communicable diseases in Japan. *Int J Infect Dis*. 2023;128:265-271. doi:10.1016/j.ijid.2023.01.013

42. Hsu HT, Huang FL, Ting PJ, Chang CC, Chen PY. The epidemiological features of pediatric viral respiratory infection during the COVID-19 pandemic in Taiwan. *Journal of microbiology, immunology, and infection = Wei mian yu gan ran za zhi*. 2021. doi:10.1016/j.jmii.2021.09.017

43. Hu CY, Tang YW, Su QM, et al. Public Health Measures During the COVID-19 Pandemic Reduce the Spread of Other Respiratory Infectious Diseases. *Front Public Health*. 2021;9:771638. doi:10.3389/fpubh.2021.771638

44. Hu W, Fries AC, DeMarcus LS, et al. Circulating Trends of Influenza and Other Seasonal Respiratory Viruses among the US Department of Defense Personnel in the United States: Impact of the COVID-19 Pandemic. *Int J Environ Res Public Health*. 2022;19(10). doi:10.3390/ijerph19105942

45. Huang QM, Song WQ, Liang F, et al. Non-Pharmaceutical Interventions Implemented to Control the COVID-19 Were Associated With Reduction of Influenza Incidence. *Front Public Health*. 2022;10:773271. doi:10.3389/fpubh.2022.773271

46. Hwang SH, Lee H, Jung M, et al. Incidence, Severity, and Mortality of Influenza During 2010-2020 in Korea: A Nationwide Study Based on the Population-Based National Health Insurance Service Database. *J Korean Med Sci*. 2023;38(8):e58. doi:10.3346/jkms.2023.38.e58

47. Ippolito G, La Vecchia A, Umbrello G, et al. Disappearance of Seasonal Respiratory Viruses in Children Under Two Years Old During COVID-19 Pandemic: A Monocentric Retrospective Study in Milan, Italy. *Frontiers in Pediatrics*. 2021;9. doi:10.3389/fped.2021.721005

48. Izu A, Nunes MC, Solomon F, et al. All-cause and pathogen-specific lower respiratory tract infection hospital admissions in children younger than 5 years during the COVID-19 pandemic (2020-22) compared with the pre-pandemic period (2015-19) in South Africa: an observational study. *Lancet Infect Dis*. 2023;23(9):1031-1041. doi:10.1016/s1473-3099(23)00200-1

49. Jayaram A, Jagadesh A, Kumar AMV, et al. Trends in Influenza Infections in Three States of India from 2015-2021: Has There Been a Change during COVID-19 Pandemic? *Trop Med Infect Dis*. 2022;7(6). doi:10.3390/tropicalmed7060110

50. Kadambari S, Goldacre R, Morris E, Goldacre MJ, Pollard AJ. Indirect effects of the covid-19 pandemic on childhood infection in England: population based observational study. *Bmj*. 2022;376:e067519. doi:10.1136/bmj-2021-067519

51. Kanda N, Hashimoto H, Imai T, et al. Indirect impact of the COVID-19 pandemic on the incidence of non-COVID-19 infectious diseases: a region-wide, patient-based database study in Japan. *Public Health*. 2023;214:20-24. doi:10.1016/j.puhe.2022.10.018

52. Kim HM, Rhee JE, Lee NJ, et al. Recent increase in the detection of human parainfluenza virus during the coronavirus disease-2019 pandemic in the Republic of Korea. *Virol J*. 2022;19(1):215. doi:10.1186/s12985-022-01938-4

53. Knudsen PK, Lind A, Klundby I, Dudman S. The incidence of infectious diseases and viruses other than SARS-CoV-2 amongst hospitalised children in Oslo, Norway during the Covid-19 pandemic 2020–2021. *Journal of Clinical Virology Plus*. 2022;2(1):100060. doi:10.1016/j.jcvp.2021.100060

54. Kume Y, Hashimoto K, Chishiki M, et al. Changes in virus detection in hospitalized children before and after the severe acute respiratory syndrome coronavirus 2 pandemic. *Influenza and Other Respiratory Viruses*. 2022;doi:10.1111/irv.12995

55. Lampros A, Talla C, Diarra M, et al. Shifting Patterns of Influenza Circulation during the COVID-19 Pandemic, Senegal. *Emerg Infect Dis*. 2023;29(9):1808-1817. doi:10.3201/eid2909.230307

56. Lamrani Hanchi A, Guennouni M, Ben Houmich T, et al. Changes in the Epidemiology of Respiratory Pathogens in Children during the COVID-19 Pandemic. *Pathogens*. 2022;11(12). doi:10.3390/pathogens11121542

57. Lee H, Lee H, Song KH, et al. Impact of Public Health Interventions on Seasonal Influenza Activity During the COVID-19 Outbreak in Korea. *Clin Infect Dis*. 2021;73(1):e132-e140. doi:10.1093/cid/ciaa672

58. Leng MD. A trend for decrease of influenza infections in children during the first wave of COVID-19 observed in a Chinese hospital. *Journal of Laboratory Medicine*. 2021;45(4-5):241-243. doi:10.1515/labmed-2021-0069

59. Li L, Wang H, Liu A, et al. Comparison of 11 respiratory pathogens among hospitalized children before and during the COVID-19 epidemic in Shenzhen, China. *Virology Journal*. 2021;18:202. doi:10.1186/s12985-021-01669-y

60. Li F, Zhang Y, Shi P, et al. Epidemiology of Viruses Causing Pediatric Community Acquired Pneumonia in Shanghai During 2010-2020: What Happened Before and After the COVID-19 Outbreak? *Infect Dis Ther*. 2022;11(1):165-174. doi:10.1007/s40121-021-00548-x

61. Liu P, Xu M, Cao L, et al. Impact of COVID-19 pandemic on the prevalence of respiratory viruses in children with lower respiratory tract infections in China. *Virology Journal*. 2021;18(1):159. doi:10.1186/s12985-021-01627-8

62. Liu P, Xu M, Lu L, et al. The changing pattern of common respiratory and enteric viruses among outpatient children in Shanghai, China: Two years of the COVID-19 pandemic. *Journal of Medical Virology*. 2022;doi:10.1002/jmv.27896

63. Liu R-h, Zhang Y-y, Lu Z-h, et al. Impact of COVID-19 pandemic on the etiology and characteristics of community-acquired pneumonia among children requiring bronchoalveolar lavage in northern China. medRxiv; 2023.

64. Liu P, Xu J. Resurgence of influenza virus activity during COVID-19 pandemic in Shanghai, China. *J Infect*. 2023;86(1):66-117. doi:10.1016/j.jinf.2022.09.025

65. Lu Y, Wang Y, Shen C, Luo J, Yu W. Decreased Incidence of Influenza During the COVID-19 Pandemic. *Int J Gen Med*. 2022;15:2957-2962. doi:10.2147/ijgm.S343940

66. Lumley SF, Richens N, Lees E, et al. Changes in paediatric respiratory infections at a UK teaching hospital 2016-2021; impact of the SARS-CoV-2 pandemic. *J Infect*. 2022;84(1):40-47. doi:10.1016/j.jinf.2021.10.022

67. Łuniewska K, Szymański K, Kondratiuk K, Hallmann E, Brydak LB. The Impact of the COVID-19 Pandemic on Influenza Transmission in Poland. *Microorganisms*. 2023;11(4). doi:10.3390/microorganisms11040970

68. Luo Z, Li S, Li N, et al. Assessment of Pediatric Outpatient Visits for Notifiable Infectious Diseases in a University Hospital in Beijing During COVID-19. *JAMA Netw Open*. 2020;3(8):e2019224. doi:10.1001/jamanetworkopen.2020.19224

69. Maison N, Peck A, Illi S, et al. The rising of old foes: impact of lockdown periods on "non-SARS-CoV-2" viral respiratory and gastrointestinal infections. *Infection*. 2022;50(2):519-524. doi:10.1007/s15010-022-01756-4

70. Márquez Caballero J, Cordero Matía ME. Epidemiology of Acute Bronchiolitis in a Third-level Hospital During the COVID-19 Pandemic. *Arch Bronconeumol*. 2023;59(4):264-266. doi:10.1016/j.arbres.2022.11.002

71. Maruo Y, Ishikawa S, Oura K, et al. The impact of the coronavirus disease 2019 pandemic on pediatric hospitalization in Kitami, Japan. *Pediatrics international : official journal of the Japan Pediatric Society*. 2022;64(1):e14937. doi:10.1111/ped.14937

72. Moscovich DP, Averbuch D, Kerem E, et al. Pediatric respiratory admissions and related viral infections during the COVID-19 pandemic. *Pediatr Pulmonol*. 2023;58(7):2076-2084. doi:10.1002/ppul.26434

73. Nott R, Fuller TL, Brasil P, Nielsen-Saines K. Out-of-Season Influenza during a COVID-19 Void in the State of Rio de Janeiro, Brazil: Temperature Matters. *Vaccines (Basel)*. 2022;10(5). doi:10.3390/vaccines10050821

74. Nwosu A, Lee L, Schmidt K, Buckrell S, Sevenhuysen C, Bancej C. National Influenza Annual Report, Canada, 2020-2021, in the global context. *Can Commun Dis Rep*. 2021;47(10):405-413. doi:10.14745/ccdr.v47i10a02

75. Park KY, Seo S, Han J, Park JY. Respiratory virus surveillance in Canada during the COVID-19 pandemic: An epidemiological analysis of the effectiveness of pandemic-related public health measures in reducing seasonal respiratory viruses test positivity. *PLoS One*. 2021;16(6):e0253451. doi:10.1371/journal.pone.0253451

76. Parry MF, Shah AK, Sestovic M, Salter S. Precipitous Fall in Common Respiratory Viral Infections During COVID-19. *Open Forum Infect Dis*. 2020;7(11):ofaa511. doi:10.1093/ofid/ofaa511

77. Perez A, Lively JY, Curns A, et al. Respiratory Virus Surveillance Among Children with Acute Respiratory Illnesses - New Vaccine Surveillance Network, United States, 2016-2021. *MMWR Morb Mortal Wkly Rep*. 2022;71(40):1253-1259. doi:10.15585/mmwr.mm7140a1

78. Pierce A, Haworth-Brockman M, Marin D, Rueda ZV, Keynan Y. Changes in the incidence of seasonal influenza in response to COVID-19 social distancing measures: an observational study based on Canada's national influenza surveillance system. *Can J Public Health*. 2021;112(4):620-628. doi:10.17269/s41997-021-00509-4

79. Polcwiartek LB, Polcwiartek C, Andersen MP, et al. Consequences of coronavirus disease-2019 (COVID-19) lockdown on infection-related hospitalizations among the pediatric population in Denmark. 2021;180(6):1955-1963. doi:10.1007/s00431-021-03934-2

80. Razanajatovo NH, Randriambolamanantsoa TH, Rabarison JH, et al. Epidemiological Patterns of Seasonal Respiratory Viruses during the COVID-19 Pandemic in Madagascar, March 2020-May 2022. *Viruses*. 2022;15(1). doi:10.3390/v15010012

81. Ren L, Lin L, Zhang H, et al. Epidemiological and clinical characteristics of respiratory syncytial virus and influenza infections in hospitalized children before and during the COVID-19 pandemic in Central China. *Influenza Other Respir Viruses*. 2023;17(2):e13103. doi:10.1111/irv.13103

82. Sachs N, Goldberg L, Levinsky Y, et al. The Effect of the COVID-19 Pandemic on Pediatric Respiratory Hospitalizations. *Isr Med Assoc J*. 2023;25(3):171-176.

83. Sakamoto H, Ishikane M, Ueda P. Seasonal Influenza Activity During the SARS-CoV-2 Outbreak in Japan. *Jama*. 2020;doi:10.1001/jama.2020.6173

84. Sberna G, Lalle E, Valli MB, Bordi L, Garbuglia AR, Amendola A. Changes in the Circulation of Common Respiratory Pathogens among Hospitalized Patients with Influenza-like Illnesses in the Lazio Region (Italy) during Fall Season of the Past Three Years. *Int J Environ Res Public Health*. 2022;19(10). doi:10.3390/ijerph19105962

85. Serigstad S, Markussen DL, Ritz C, et al. The changing spectrum of microbial aetiology of respiratory tract infections in hospitalized patients before and during the COVID-19 pandemic. *BMC Infect Dis*. 2022;22(1):763. doi:10.1186/s12879-022-07732-5

86. Shi HJ, Kim NY, Eom SA, et al. Effects of Non-Pharmacological Interventions on Respiratory Viruses Other Than SARS-CoV-2: Analysis of Laboratory Surveillance and Literature Review From 2018 to 2021. *J Korean Med Sci*. 2022;37(21):e172. doi:10.3346/jkms.2022.37.e172

87. Shi T, Zhang X, Meng L, et al. Immediate and long-term changes in infectious diseases in China at the "First-level-response", "Normalized-control" and "Dynamic-COVID-zero" stages from 2020 to 2022: a multistage interrupted-time-series-analysis. *BMC Public Health*. 2023;23(1):1381. doi:10.1186/s12889-023-16318-y

88. Shichijo K, Takeuchi S, Tayama T, et al. Patient attendance at a pediatric emergency referral hospital in an area with low COVID-19 incidence. *PLoS One*. 2021;16(10). doi:10.1371/journal.pone.0258478

89. Song S, Li Q, Shen L, et al. From Outbreak to Near Disappearance: How Did Non-pharmaceutical Interventions Against COVID-19 Affect the Transmission of Influenza Virus? *Front Public Health*. 2022;10:863522. doi:10.3389/fpubh.2022.863522

90. Stamm P, Sagoschen I, Weise K, et al. Influenza and RSV incidence during COVID-19 pandemic-an observational study from in-hospital point-of-care testing. *Medical Microbiology and Immunology*. 2021;210(5):277-282. doi:10.1007/s00430-021-00720-7

91. Steinfort DP, Cowie B, Johnson DF. Impact of general social distancing measures on incidence of influenza in Australia. *ERJ Open Res*. 2020;6(4). doi:10.1183/23120541.00507-2020

92. Sullivan SG, Carlson S, Cheng AC, et al. Where has all the influenza gone? The impact of COVID-19 on the circulation of influenza and other respiratory viruses, Australia, March to September 2020. *Euro Surveill*. 2020;25(47). doi:10.2807/1560-7917.Es.2020.25.47.2001847

93. Suntronwong N, Thongpan I, Chuchaona W, et al. Impact of COVID-19 public health interventions on influenza incidence in Thailand. *Pathog Glob Health*. 2020:1-3. doi:10.1080/20477724.2020.1777803

94. Tan J, Liang L, Huang P, et al. Changes in Influenza Activities Impacted by NPI Based on 4-Year Surveillance in China: Epidemic Patterns and Trends. *J Epidemiol Glob Health*. 2023;doi:10.1007/s44197-023-00134-z

95. Tang HJ, Lai CC, Chao CM. The Collateral Effect of COVID-19 on the Epidemiology of Airborne/Droplet-Transmitted Notifiable Infectious Diseases in Taiwan. *Antibiotics (Basel)*. 2022;11(4). doi:10.3390/antibiotics11040478

96. Teutsch SM, Nunez CA, Morris A, et al. Australian Paediatric Surveillance Unit (APSU) Annual Surveillance Report 2020. *Commun Dis Intell (2018)*. 2021;45doi:10.33321/cdi.2021.45.59

97. Trenholme A, Webb R, Lawrence S, et al. COVID-19 and Infant Hospitalizations for Seasonal Respiratory Virus Infections, New Zealand, 2020. *Emerging Infectious Diseases*. 2021;27(2):641-643. doi:10.3201/eid2702.204041

98. Uhteg K, Amadi A, Forman M, Mostafa HH. Circulation of Non-SARS-CoV-2 Respiratory Pathogens and Coinfection with SARS-CoV-2 Amid the COVID-19 Pandemic. *Open Forum Infect Dis*. 2022;9(3):ofab618. doi:10.1093/ofid/ofab618

99. Venkatram S, Alapati A, Dileep A, Diaz-Fuentes G. Change in patterns of hospitalization for influenza during COVID-19 surges. *Influenza Other Respir Viruses*. 2022;16(1):72-78. doi:10.1111/irv.12900

100. Vila J, Lera E, Andrés C, et al. The burden of non-SARS-CoV2 viral lower respiratory tract infections in hospitalized children in Barcelona (Spain): A long-term, clinical, epidemiologic and economic study. *Influenza Other Respir Viruses*. 2023;17(1):e13085. doi:10.1111/irv.13085

101. Vittucci AC, Piccioni L, Coltella L, et al. The Disappearance of Respiratory Viruses in Children during the COVID-19 Pandemic. *International Journal of Environmental Research and Public Health*. 2021;18(18). doi:10.3390/ijerph18189550

102. Wagatsuma K, Koolhof IS, Saito R. Was the Reduction in Seasonal Influenza Transmission during 2020 Attributable to Non-Pharmaceutical Interventions to Contain Coronavirus Disease 2019 (COVID-19) in Japan? *Viruses*. 2022;14(7)doi:10.3390/v14071417

103. Wang J, Xiao T, Xiao F, et al. Time Distributions of Common Respiratory Pathogens Under the Spread of SARS-CoV-2 Among Children in Xiamen, China. *Frontiers in Pediatrics*. 2021;9:584874. doi:10.3389/fped.2021.584874

104. Wang B, Gai X, Han Y, et al. Epidemiological characteristics of common respiratory infectious diseases in children before and during the COVID-19 epidemic. *Front Pediatr*. 2023;11:1212658. doi:10.3389/fped.2023.1212658

105. Wu K, Wu X, Wang W, Hong L. Epidemiology of influenza under the coronavirus disease 2019 pandemic in Nanjing, China. *J Med Virol*. 2022;94(5):1959-1966. doi:10.1002/jmv.27553

106. Xiao J, Dai J, Hu J, et al. Co-benefits of nonpharmaceutical intervention against COVID-19 on infectious diseases in China: A large population-based observational study. *Lancet Reg Health West Pac*. 2021;17:100282. doi:10.1016/j.lanwpc.2021.100282

107. Xu M, Liu P, Su L, et al. Comparison of Respiratory Pathogens in Children With Lower Respiratory Tract Infections Before and During the COVID-19 Pandemic in Shanghai, China. *Front Pediatr*. 2022;10:881224. doi:10.3389/fped.2022.881224

108. Ye Q, Wang D. Epidemiological changes of common respiratory viruses in children during the COVID-19 pandemic. *Journal of Medical Virology*. 2022;94(5):1990-1997. doi:10.1002/jmv.27570

109. Ye Q, Liu H, Mao J, Shu Q. Nonpharmaceutical interventions for COVID-19 disrupt the dynamic balance between influenza A virus and human immunity. *J Med Virol*. 2023;95(1):e28292. doi:10.1002/jmv.28292

110. Yeoh DK, Foley DA, Minney-Smith CA, et al. Impact of Coronavirus Disease 2019 Public Health Measures on Detections of Influenza and Respiratory Syncytial Virus in Children During the 2020 Australian Winter. *Clinical infectious diseases : an official publication of the Infectious Diseases Society of America*. 2021;72(12):2199-2202. doi:10.1093/cid/ciaa1475

111. Yi S, Zhang WX, Zhou YG, et al. Epidemiological change of influenza virus in hospitalized children with acute respiratory tract infection during 2014-2022 in Hubei Province, China. *Virol J*. 2023;20(1):122. doi:10.1186/s12985-023-02092-1

112. Yorsaeng R, Suntronwong N, Thongpan I, et al. The impact of COVID-19 and control measures on public health in Thailand, 2020. *PeerJ*. 2022;10:e12960. doi:10.7717/peerj.12960

113. Zhang J, Yang T, Zou M, Wang L, Sai L. The epidemiological features of respiratory tract infection using the multiplex panels detection during COVID-19 pandemic in Shandong province, China. *Sci Rep*. 2023;13(1):6319. doi:10.1038/s41598-023-33627-9

114. Zheng L, Qi J, Wu J, Zheng M. Changes in Influenza Activity and Circulating Subtypes During the COVID-19 Outbreak in China. *Front Med (Lausanne)*. 2022;9:829799. doi:10.3389/fmed.2022.829799

115. Zuo Z, Yang C, Ye F, et al. Trends in respiratory diseases before and after the COVID-19 pandemic in China from 2010 to 2021. *BMC Public Health*. 2023;23(1):217. doi:10.1186/s12889-023-15081-4
